# Supplementary material for: Motility phenotype in a zebrafish vmat2 mutant
Source: PLoS One. 2022 Jan 5;17(1):e0259753. doi: 10.1371/journal.pone.0259753 (PMC8730441; doi:10.1371/journal.pone.0259753)
Supplement: Attachment — Submitted filename: Reply to reviewers.docx [file pone.0259753.s002.docx]

Dear Editor. What follows is a complete list of the changes made in the manuscript. First, the checklist provided by PLoS and, second, the specific comments raised by the reviewers.

1. Plos Style policy: *The manuscript now adheres to PLoS style policy.*
2. Funding information: *I have verified that the grant numbers as stated in the manuscript are correct:* GM067841 (RAC) *and* AR062547 (RAC). *And the funding statements in the submission format and manuscript now match.*
3. Competing interests statment: *The correct statement is now in the manuscript.*
4. Data availability: *All data used in the paper is now submitted as S1.*
5. Data not shown: *The phrase „data not shown“ has been removed. Now we simply state „N.S.“ since 0.05 has been defined as the significance level elsewhere in the manuscript. The relevant data is of course stored with full data set (see comment 4 above).*
6. Reference section: *References 49-54 have been added to the reference section (see comment a) from Reviewer #2).*
7. Reviewer #1 comments:
   a. Order of paragraphs P10: *The order of the paragraphs have been changed to match the order of panels in Figure 1.*
   b. IHC pictures: *We feel that the IHC pictures are of sufficient quality to convey the key message of reduced DA cell count.*
   c. Line 87: typo: *Fixed*.
   d. Line 338: typo: *Fixed*
8. Reviewer #2 comments:
   a. Contrast to other PD models: *We added a short paragraph on comparison to other genetic PD models, and the appropriate references, to the fourth paragraph of the discussion. A full comparison of all zebrafish PD models is beyond the scope of this paper as can be seen in reference #49.*
   b. MPTP/MPP+ additional experiment: *The reviewer raises an interesting point. In fact, a subset of the authors have performed this experiment, as a part of seperate study that we cannot add to this one. But, the reviewer was indeed onto something since the vmat2 mutants seem to be hypersensitive to MPP+.*

We feel that we have fully and completely addressed all questions, comments and suggestions made. We thank the reviewers for their contribution in improving our manuscript.

Dr. Karl Ægir Karlsson

Professor
Biomedical Engineering
School of Science and Engineering
Reykjavik University
Menntavegur 1, 101, Reykjavik, Iceland
Tel: +(354) 599 6467
Cell: +(354) 825 6467
E-mail: [karlsson@ru.is](mailto:karlsson@ru.is)
